# Safety evaluation of outpatient vs inpatient unicompartmental knee arthroplasty: a systematic review and meta-analysis

**DOI:** 10.1007/s00402-024-05446-8

**Published:** 2024-07-13

**Authors:** Jia-Wang Zhu, Tong-Fu Wang, De-Sheng Chen, Lei Lei

**Affiliations:** Department of Sports Medicine and Arthroscopy, Tianjin Hospital of Tianjin University, Tianjin, China

**Keywords:** Unicompartmental knee arthroplasty, Outpatient, Inpatient, Safety

## Abstract

**Purpose:**

This systematic review and meta-analysis aimed to evaluate the safety of outpatient and inpatient Unicompartmental Knee Arthroplasty (UKA) based on the incidence of adverse events.

**Method:**

A systematic search of the literature was performed in October 2022 on PubMed, Web of Science, Cochrane library, and Embase. The Meta package for R was used to perform the meta-analysis.

**Result:**

Five studies with a total of 26,301 patients were included. 5813 patients (22.1%) were treated with outpatient UKA, and 20,488 patients (77.9%) were treated with inpatient UKA. There were no statistically significant differences in the incidence of total complications (RR = 1.36, 95% CI = 0.64–2.89, Z = 0.79, P = 0.43), readmission (RR = 1.02, 95% CI = 0.40–2.60, Z = 0.05, P = 0.96), and venous thrombosis (RR = 1.43, 95% CI = 0.96–2.11, Z = 1.78, P = 0.08). Incidence rates were lower in outpatient UKA regarding urinary tract infection (RR = 1.48, 95% CI = 1.07–2.04, Z = 2.40, P = 0.02), pulmonary embolus (RR = 7.48, 95% CI = 1.80–31.17, Z = 2.76, P < 0.01), and transfusion (RR = 2.77, 95% CI = 1.63–4.71, Z = 3.78, P < 0.01).

**Conclusion:**

In summary, outpatient UKA shows lower incidences of hospital-acquired complications such urinary tract infection, pulmonary embolus, and transfusion. It's worth noting that the incidences of total complications, readmission, and venous thrombosis in outpatient UKA were not higher than the incidences of inpatient UKA, suggestting that outpatient UKA can be considered a safe alternative to inpatient UKA.

## Introduction

Unicompartmental knee arthroplasty (UKA) is becoming an increasingly popular procedure for unicompartmental knee osteoarthritis [1–3]. Traditionally, UKA was considered an inpatient procedure, while recently several studies have reported that outpatient UKA is feasible [4–7].

Outpatient UKA is considered beneficial to reduce the risk of hospital-acquired infections, start early rehabilitation in a familiar environment, and improve patient satisfaction, besides, outpatient UKA can be an attractive option to reduce the economic burden on the healthcare systems [8–11].

It is unclear which of these two procedures is clinically superior and safer. The purpose of this study was to synthesize the current literature and provide further data regarding the safety of outpatient and inpatient UKA.

## Materials and methods

According to PRISMA guidelines, this meta-analysis was conducted.

### Search strategy

Potentially relevant articles were identified through searches in PubMed, Web of Science, Cochrane library, Embase, and Google Scholar from inception to October 2022. The keywords “unicompartmental arthroplasty”, “outpatient” and “inpatient” combined using Boolean logic were used to conduct the search. The reference lists of retrieved articles were also searched for additional studies.

### Inclusion criteria

Studies were considered eligible if they met all of the following criteria: (1) the patients were treated with primary UKA; (2) The study was designed to compare outcomes of outpatient and inpatient UKA; (3) reporting at least one of the following outcomes: total complications, readmission, urinary tract infection, pulmonary embolism, venous thrombosis, and erythrocyte transfusion. Total complications were defined as various adverse events occurring in patients postoperatively, such as surgical complications, infections, bleeding, nerve injuries, and others.

### Exclusive criteria

The following articles were excluded from the search: (1) duplicate articles or articles with overlapping data; (2) case reports, conference reports, systematic reviews, and meta-analyses; (3) article is not written in English.

### Data extraction

A pair of independent reviewers screened titles/abstracts and relevant full-text studies, in case of disagreement, a third investigator was consulted. The following information was extracted from studies: the author’s name, year of publication, study type, patient demographics, and the indicators of adverse events, including total complications, readmission, urinary tract infection, pulmonary embolism, venous thrombosis, and transfusion.

### Quality assessment

The quality of the study was assessed using the Newcastle–Ottawa Scale (NOS). For each observational study, a score of 0–9 was assigned. A study with a score > 6 was considered high quality.

### Data analysis and statistical methods

The statistical analysis was performed using the R package “meta”. The I^2^ test and the Q test were used to assess heterogeneity between the included studies. If there was heterogeneity (p < 0.05 or I^2^ > 50%), we used a random-effects model, otherwise, we used a fixed-effects model. We presented mean differences (MDs) and 95% confidence intervals (CIs) for continuous outcomes. For dichotomous variables, risk ratios (RRs) with 95% CIs were calculated.

## Results

### Search results

**Fig. 1:**
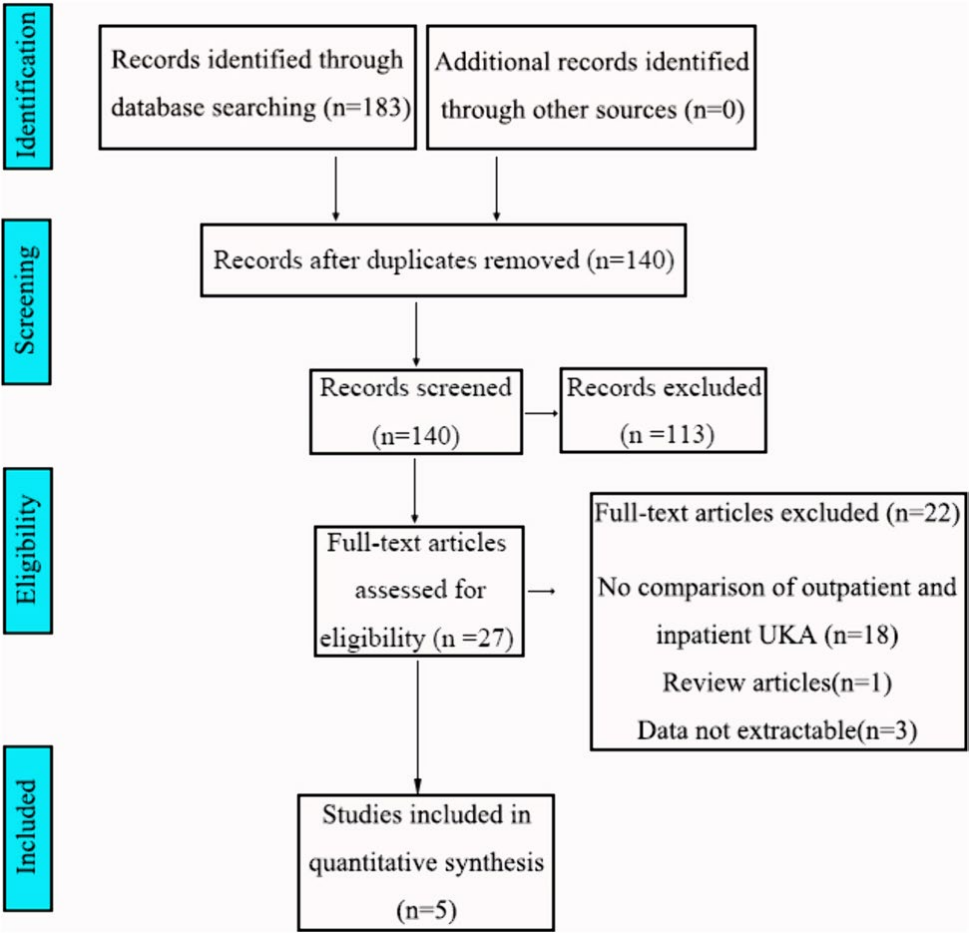
PRISMA Flow Chart of the study selection process

A total of 183 studies were identified through the search strategy. After removing duplicates, 140 studies remained. Then, a total of 113 studies were excluded based on their titles and abstracts. Eventually, 5 studies were eligible for data extraction and meta-analysis (Fig. 1).

### Characteristics of the included studies

A total of 26,301 patients were included in our analysis. 5813 patients (22.1%) were treated with outpatient UKA, and 20,488 patients (77.9%) were treated with inpatient UKA. Patient demographic characteristics are presented in Table 1.

**Table 1:** Summary of included study characteristics and demographics

Author	Year	Country	Type	Patients (n)		Females (n)		Age (Years)		NOS score
				Inpatient	Outpatient	Inpatient	Outpatient	Inpatient	Outpatient	
Dustin [8]	2017	USA	Retrospective cohort	10	12	2 (20%)	5 (42%)	64.5	67.2	6
Bovonratwet [20]	2017	USA	Retrospective cohort	5312	568	2811 (52.9%)	284 (50.0%)	63.7	62.9	8
Marcus [7]	2020	USA	Retrospective cohort	48	48	28 (58.3%)	33 (68.7%)	59.4	58.8	6
Liam [6]	2020	USA	Retrospective cohort	5940	3181	3231 (54.4%)	1720 (54.1%)	NA	NA	7
Edward [19]	2021	USA	Retrospective cohort	9178	2004	4812 (52.4%)	937 (46.8%)	NA	NA	7

### Total complications

**Fig. 2:**
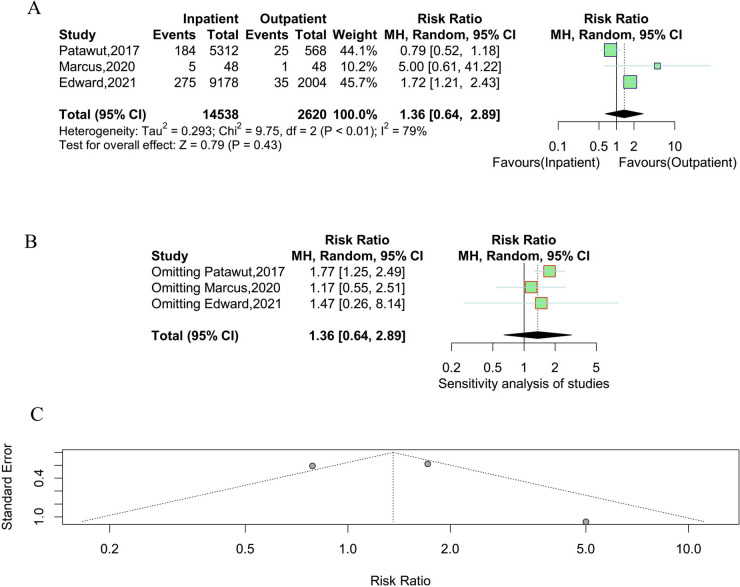
Result of the incidence of total complications between outpatient and inpatient UKA. **A** forest plot for meta-analysis; **B** forest plot for sensitivity analysis; **C** funnel plot for publication bias

Three studies were enrolled to compare the incidence of total complications between outpatient and inpatient UKA. As shown in Table 2, significant heterogeneity existed among studies and a random effect model was employed (P < 0.01, I^2^ = 79%). As shown in Fig. 2, there were no statistically significant differences in the incidence rate of total complications between outpatient and inpatient UKA. The results of the sensitivity analysis suggested that the result was unstable. Meanwhile, there was no obvious publication bias observed in the funnel plot and Egger’s test (t = 0.30, P = 0.82).

**Table 2:** Adverse events summary and assessment

Adverse events	RR	95% CI	Z-value	P-value	Sensitivity Analysis	Publication Bias	Significant heterogeneity	Random / Fixed effect model
Total Complications	1.36	0.64–2.89	0.79	0.43	Unstable	Not observed	Yes	Random
Readmission	1.02	0.40–2.60	0.05	0.96	Unstable	Not observed	Yes	Random
Venous thrombosis	1.43	0.96–2.11	1.78	0.08	Unstable	Not observed	No	Fixed
Urinary tract infection	1.48	1.07–2.04	2.40	0.02	Stable	Not observed	No	Fixed
Pulmonary embolus	7.48	1.80–31.17	2.76	< 0.01	Stable	Not observed	No	Fixed

### Readmission

**Fig. 3:**
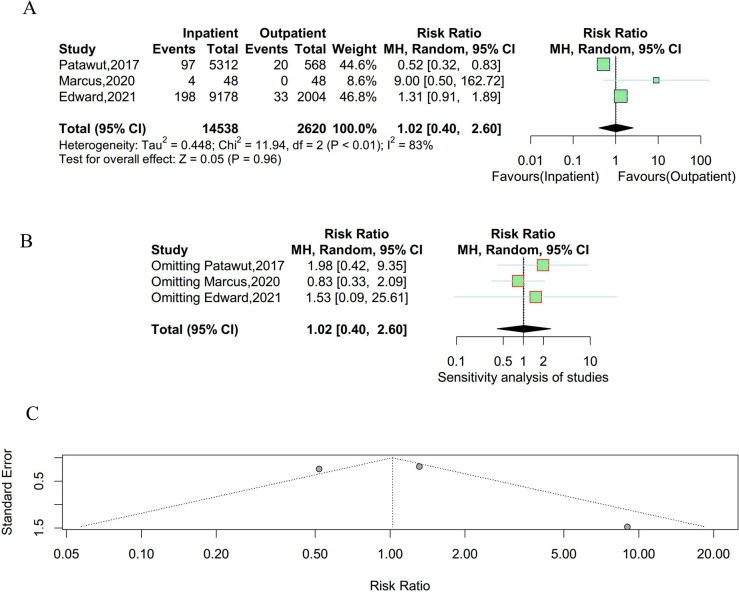
Result of the incidence of readmission between outpatient and inpatient UKA. **A** forest plot for meta-analysis; **B** forest plot for sensitivity analysis; **C** funnel plot for publication bias

Three studies were enrolled to compare the incidence of readmission between outpatient and inpatient UKA. Significant heterogeneity existed among studies and a random effect model was employed (P < 0.01, I^2^ = 83%). As shown in Fig. 3, there were no statistically significant differences in the incidence rate of readmission between outpatient and inpatient UKA. The results of the sensitivity analysis suggested that the result was unstable. Meanwhile, there was no obvious publication bias observed in the funnel plot and Egger’s test (t = 0.26, P = 0.84).

### Venous thrombosis

**Fig. 4:**
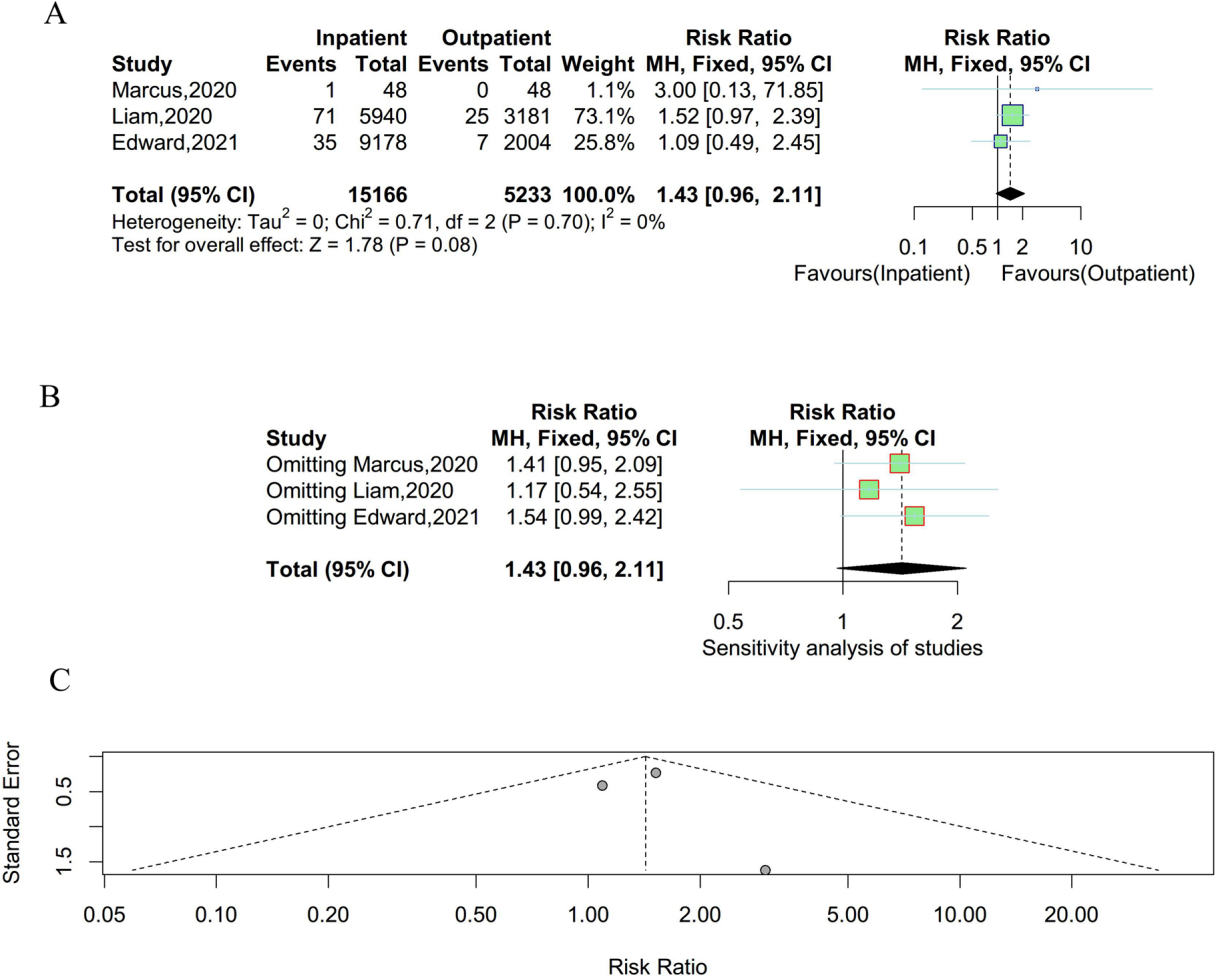
Result of the incidence of venous thrombosis between outpatient and inpatient UKA. **A** forest plot for meta-analysis; **B** forest plot for sensitivity analysis; **C** funnel plot for publication bias

Three studies were enrolled to compare the incidence of venous thrombosis between outpatient and inpatient UKA. There was no significant heterogeneity existing among studies and a fixed effect model was employed (P = 0.70, I^2^ = 0%). As shown in Fig. 4, there were no statistically significant differences in the incidence rate of venous thrombosis between outpatient and inpatient UKA. The results of the sensitivity analysis suggested that the result was unstable. Meanwhile, there was no obvious publication bias observed in the funnel plot and Egger’s test (t = 0.15, P = 0.90).

### Urinary tract infection

**Fig. 5:**
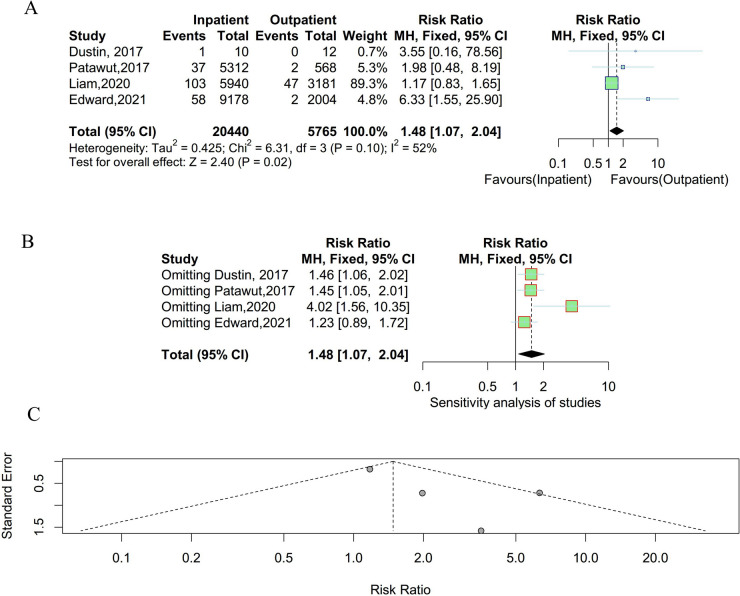
Result of the incidence of urinary tract infection between outpatient and inpatient UKA. **A** forest plot for meta-analysis; **B** forest plot for sensitivity analysis; **C** funnel plot for publication bias

Four studies were enrolled to compare the incidence of urinary tract infection between outpatient and inpatient UKA. There was no significant heterogeneity existing among studies and a fixed effect model was employed (P = 0.10, I^2^ = 52%). As shown in Fig. 5, the incidence rate of urinary tract infection was lower in outpatient. The results of the sensitivity analysis suggested that the result was stable and reliable. Meanwhile, there was no obvious publication bias observed in the funnel plot and Egger’s test (t = 1.96, P = 0.19).

### Pulmonary embolus

**Fig. 6:**
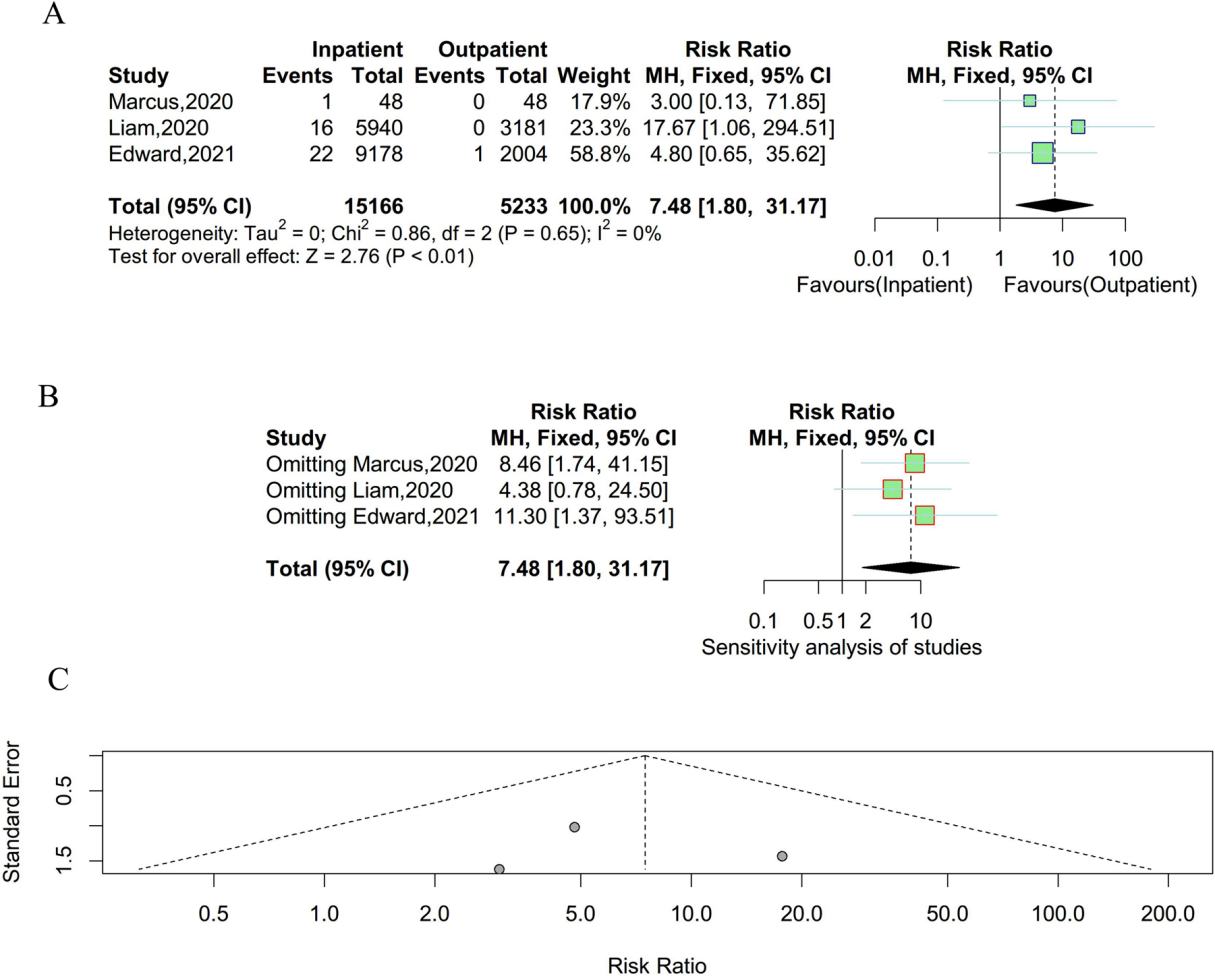
Result of the incidence of pulmonary embolus between outpatient and inpatient UKA. **A** forest plot for meta-analysis; **B** forest plot for sensitivity analysis; **C** funnel plot for publication bias

Three studies were enrolled to compare the incidence of pulmonary embolus between outpatient and inpatient UKA. There was no significant heterogeneity existing among studies and a fixed effect model was employed (P = 0.65, I^2^ = 0%). As shown in Fig. 6, the incidence rate of pulmonary embolus was lower in outpatient. The results of the sensitivity analysis suggested that the result was stable and reliable. Meanwhile, there was no obvious publication bias observed in the funnel plot and Egger’s test (t = 0.16, P = 0.90).

### Transfusion

**Fig. 7:**
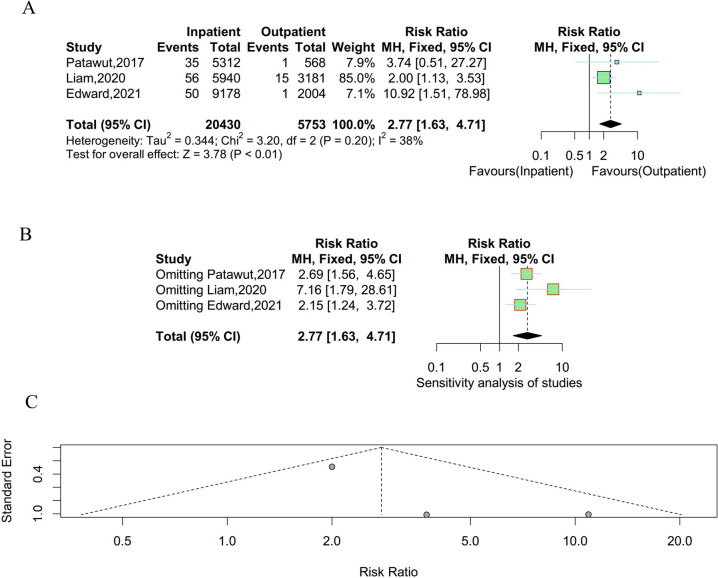
Result of the incidence of transfusion between outpatient and inpatient UKA. **A** forest plot for meta-analysis; **B** forest plot for sensitivity analysis; **C** funnel plot for publication bias

Three studies were enrolled to compare the incidence of transfusion between outpatient and inpatient UKA. There was no significant heterogeneity existing among studies and a fixed effect model was employed (P = 0.20, I^2^ = 38%). As shown in Fig. 7, the incidence rate of transfusion was lower in outpatient. The results of the sensitivity analysis suggested that the result was stable and reliable. Meanwhile, there was no obvious publication bias observed in the funnel plot and Egger’s test (t = 2.00, P = 0.29).

## Discussion

To our knowledge, this is the first systematic review and meta-analysis to evaluate the safety of outpatient UKA. Previous studies showed that outpatient total knee arthroplasty (TKA) is a safe and effective procedure, which prompted the implementation of outpatient UKA [12–14]. Our study demonstrates that outpatient UKA shows lower incidences of hospital-acquired complications and can be considered as a safe alternative to inpatient UKA.

In fact, outpatient UKA has been performed more frequently and at a lower cost in recent years [15–18]. In a study conducted by Bosch et al, the PearlDiver database was employed and a total of 9121 patients were treated with UKA from 2007 to 2016, Meanwhile, the authors pointed out that the costs of outpatient UKA were significantly lower than the costs of inpatient UKA [6]. Similarly, Richter et al. noted that the medical costs of outpatient UKA averaged $20,500, while the medical costs of inpatient UKA averaged $46,845, in other words, almost 50% cost savings were achieved by outpatient UKA [8].

With the rising incidence of outpatient UKA, studies confirming the safety of outpatient UKA were necessary. In a study conducted by Hur et al., the patients undergoing UKA from 2005 to 2018 were reviewed, the patients were divided into an early cohort (from 2005 to 2015) and a late cohort (from 2016 to 2018), and there were lower incidence rates of surgical-site infection in inpatient UKA compared with outpatient UKA in the early cohort, in contrast, there were lower incidence rates of any complication in outpatient UKA compared with inpatient UKA in the late cohort, which indicated an improvement in quality of outpatient UKA over time [19]. Similarly, in a study conducted by Bovonratwet et al., 568 outpatient and 5312 inpatient UKA cases were reviewed, and there were no significant differences in any perioperative complications or any postoperative complications between the outpatient and inpatient cohorts [20].

In our study, outpatient UKA had lower incidences compared to inpatient UKA regarding urinary tract infection, transfusion, and pulmonary embolism. In fact, outpatient UKA may adhere to stricter patient selection criteria, primarily targeting younger individuals with fewer complications. In the study by Bovonratwet et al., the mean age of the outpatient UKA was 62.9 years, whereas the mean age of the inpatient UKA was 63.7 years [20]. Edward et al. pointed out that a higher prevalence of comorbidities such as diabetes mellitus, hypertension, chronic obstructive pulmonary disease, and hypoalbuminemia among inpatient UKA [19]. Furthermore, early ambulation and non-general anesthesia such as spinal anesthesia are frequently preferred in outpatient UKA due to the shorter hospital stay. Lei et al. pointed out that early ambulation within 24 h after TKA was associated with lower incidences of deep vein thrombosis (DVT) and pulmonary infection [21]. Although their study targeted patients with TKA, it is reasonable to expect that early ambulation has implications for complications of UKA. On the other hand, Edward et al. reported that the incidence of regional anesthesia in inpatient UKA was 32.5%, lower than the incidence of 32.9% in outpatient UKA from 2005 to 2015. Additionally, from 2016 to 2018, the incidence of regional anesthesia in inpatient UKA was 44.3%, also lower than the incidence of 46.8% in outpatient UKA [19]. Lu et al. observed that within the cohort of patients undergoing UKA, those who received general anesthesia experienced longer operative time and higher incidences of DVT and postoperative superficial infections in comparison to those who received spinal anesthesia [22]. Furtherly, Held et al. emphasized the effect of operative time on postoperative complications after UKA, they noted that surgical times longer than 2 h for primary UKA increase the risk of surgical site infection, reoperation, and blood transfusion [23].

Our study has several limitations. First, this meta-analysis only included 5 studies and the sample size of patients was relatively small, which was detrimental to strong conclusions. Second, according to the sensitivity analysis, the combined results of total complications, readmission, and venous thrombosis were unstable. Third, due to the limitations of the included studies, specific details about the duration of follow-up were not consistently reported, further studies were needed to provide insights into the issues.

In summary, outpatient UKA shows lower incidences of hospital-acquired complications such urinary tract infection, pulmonary embolus, and transfusion. It's worth noting that the incidences of total complications, readmission, and venous thrombosis in outpatient UKA were not higher than the incidences of inpatient UKA, suggestting that outpatient UKA can be considered a safe alternative to inpatient UKA.

## Data Availability

All relevant data are reported within this manuscript, additional information will be available upon request to the corresponding author.

## Code availability

All computer codes required to generate these results will be available upon request to the corresponding author.
